# Early management of sight threatening retinopathy in *incontinentia pigmenti*

**DOI:** 10.1186/s13023-020-01509-2

**Published:** 2020-08-27

**Authors:** Sarah Michel, Clothilde Reynaud, Alejandra Daruich, Smail Hadj-Rabia, Dominique Bremond-Gignac, Christine Bodemer, Matthieu P. Robert

**Affiliations:** 1 Ophthalmology Department and Rare Eye Disease Reference Center OPHTARA, Necker-Enfants malades University Hospital, AP-HP, Paris, France; 2 INSERM, UMRS1138, Team 17, From Physiopathology of Ocular Diseases to Clinical Development, Paris University, Paris, France; 3 Dermatology Department and Genodermatoses and Rare Skin Diseases Reference Center MAGEC, Necker-Enfants malades University Hospital, AP-HP, Paris, France; 4 INSERM U1163, Institut IMAGINE, Paris, France; 5 Borelli Centre, UMR 9010, CNRS-SSA-ENS Paris Saclay-Paris University, Paris, France

**Keywords:** *Incontinentia pigmenti*, Vasculopathy, Early screening, Early preventive laser therapy, Retinal detachment

## Abstract

**Background:**

Early blindness secondary to incurable retinal detachment is one of the main complications of *incontinentia pigmenti* (IP). The efficiency of ophthalmological management for preventing such evolution has not been proven.

The objective of this retrospective study was to report a screening and treatment strategy of the vascular retinopathy in newborns and infants with IP.

**Results:**

All files of patients diagnosed with IP within the two first months of life in a single tertiary referral center, between 2010 and 2015, were retrospectively included. The minimum follow-up duration was three years.

Patients had undergone systematic indirect ophthalmoscopy examination, looking for signs of peripheric retinal vasculopathy, according to a standardized schedule: at diagnosis, at age 1, 2, 3, 6, 9, 12, 18 and 24 months, and then once a year. Urgent laser therapy was performed under anesthesia in case of signs of retinal ischemia.

Nineteen children files (17 girls) were studied. Median age at IP diagnosis was 1 day [0–44]; median age at first retinal evaluation was 25 days. Retinal manifestations occurred in 7 patients (*n* = 10/38 eyes, 26.3%); they were diagnosed at median age 19 days [3–59]. These patients underwent one or two ablative session per eye (mean 1.7, median 2), under general anaesthesia. No retinal detachment or fold occurred during the follow-up (median 6 years [3–9.8]).

**Conclusion:**

Ocular screening should be performed in all cases of IP as soon as possible after diagnosis. A strict ophthalmological monitoring and prophylactic treatment of retinal vasculopathy can efficiently prevent the early blinding complications of the disease.

## Background

*Incontinentia pigmenti* (IP) is a rare X-linked dominant disease that affects the skin, eyes, central nervous system and teeth*.* The disease is due to mutations in the gene encoding the protein NEMO (NF-κB Essential Modulator), which modulates the transcription factor NF-κB [1]. This results in aberrant regulation of the transcription of multiple genes involved in immune, inflammatory and apoptotic responses. Retinal manifestations are likely due to vaso-occlusive phenomenon and inflammatory disorder. Usually lethal in male fetuses, IP predominates in women.

While the dermatological findings, usually leading to IP diagnosis within the first days of life, are clinically obvious, specific, and usually have a benign outcome, the ocular and neurological manifestations may lead to severe functional impairment [2].

Ocular manifestations are classically divided into retinal and non-retinal findings. Early acquired involvement of peripheral retinal arterioles leads to retinal ischemia and new vessels growth. If left untreated, this event sequence results in severe retinal detachment; incurable if diagnosed too late, and leading to the classical picture of retrolental fibroplasia. About 36.5% of children with IP will develop ocular abnormalities, leading to blindness in nearly 50% of affected cases [3]. Early screening and laser treatment have shown to possibly prevent such evolution [4, 5]. We therefore established, in 2010, a screening and treatment program for IP patients referred to our institution.

The objective of this study is to evaluate the efficiency of early detection and treatment of the retinal vasculopathy in infants with IP.

## Methods

Files of consecutive patients diagnosed with IP within the two first months of life at *Necker-enfants malades* hospital from 2010 to 2015 were retrospectively reviewed. The rare disease database CEMARA, in which all cases are systematically registered at diagnosis, was used for data collection. Demographic and clinical characteristics were registered at diagnosis and at each subsequent visit. Clinical characteristics included the nature and location (by quadrants: superior, temporal, inferior, and nasal) of any retinal abnormality. Anatomical outcome, with a follow-up of at least 3 years, was analyzed.

**Fig. 1:**
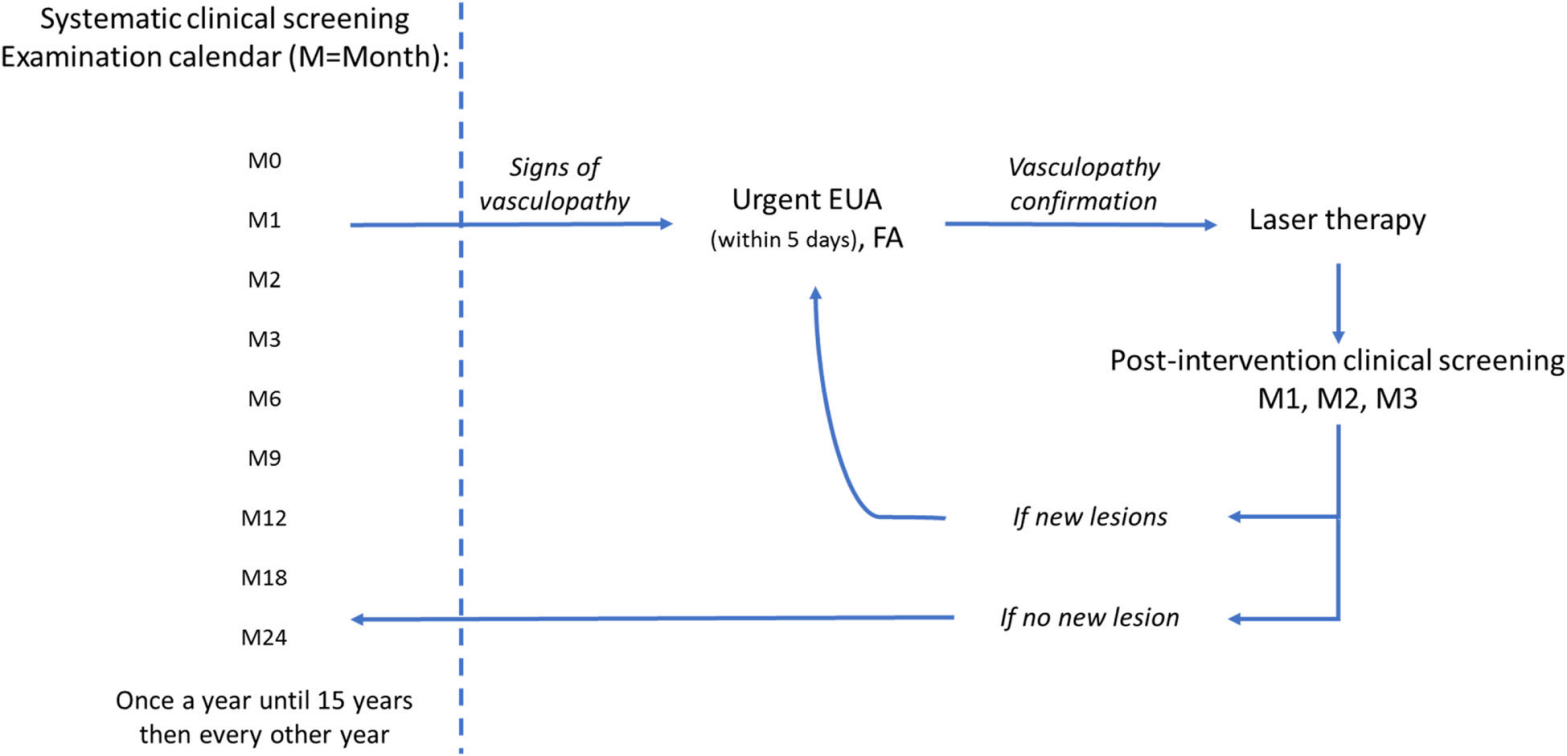
Proposed algorithm for systematic retinal screening and management in patients with *incontinentia pigmenti*. Initial fundus examination is performed in the clinic, ideally in the 48 h following diagnosis confirmation by expert dermatologist soon after birth, using indirect ophthalmoscopy, with particular attention to the periphery of the retina. It is followed by systematic examinations according to a specific calendar displayed in the figure EUA: Examination under Anesthesia. FA: Fluorescein Angiography. M = month.

Once diagnosed, patients were screened for ocular involvement by a referral ophthalmologist (SM or MPR), according to a standardized fundus monitoring schedule (Fig. 1), and laser prophylaxis was applied in case of signs of retinal ischemia.

Fundoscopy, using binocular indirect ophthalmoscope, was first performed in the clinic and then, in the presence of any abnormality compatible with a vasculopathy (vascular arrest, telangiectasia, anastomosis, neovascularization or hemorrhages), under general anesthesia, in addition to fluoresceine angiography. Non perfused retinal areas were treated by photocoagulation. Laser treatment (Quentel medical*, France) was performed using 500 μm diameter spots, up to mild whitening of the retina, as for retinopathy of prematurity followed by systematic post-treatment follow-up [6] (Fig. 1).

## Results

Nineteen children files (*n* = 38 eyes) were studied. Seventeen (89%) were girls. Patients, lesions and treatment characteristics are presented in Table 1. Retinal lesions were present in 7 patients (37%, *n* = 10 eyes) and included vascular abnormalities alone (dilation, tortuosity, anastomosis) in 4 eyes (3 patients), hemorrhages and vascular abnormalities alone in 5 eyes (4 patients) and associated with retinal new vessels in one eye. Retinal hemorrhages alone were noticed in one patient at age 21 days, and were considered banal post-delivery retinal hemorrhages; strict follow-up showed disappearance of these hemorrhages, with no other lesion. Lesions were extended to 1 to 4 quadrants (1, 2, 3, and 4 quadrants in 2, 3, 3, and 2 affected eyes, respectively). The localization of retinal lesions was: superior, temporal, inferior, and nasal in 7, 7, 7, and 4 eyes, respectively. The two boys from the series had mosaicism; both exhibited severe, bilateral retinal involvement. One or two laser sessions were performed in 7 patients (10 eyes).

**Table 1:** Clinical findings of patients with *incontinentia pigmenti.*

Included patients	19 patients (38 eyes)		
Age at incontinentia pigmenti diagnosis (days)	Mean 8.9 Median 1 Range [0-44]		
Delay between diagnosis and first ophthalmological examination (days)	Mean 5.9 Median 0 Range [0-36]		
Age at retinal disease diagnosis (days)	Mean 32.5 Median 19 Range [3-59]		
Delay between retinal disease diagnosis and examination under anesthesia (days)	Mean 9 Median 6 Range [3-28]		
Retinal manifestations: patients (eyes)	7 patients (10 eyes) (26.3%)	OD only	1 patient (1 eye)
		OS only	3 patients (3 eyes)
		OSD	3 patients (6 eyes)
Duration of follow-up (years)	Mean 6,4 Median 6,0 Range [3-9.8]		
Prophylactic ablative therapy	Treated patients	7 patients (10 eyes)	
	Ablative sessions per eye (mean)	Mean 1.7 Median 2 Range [1-2]	
	Age at first session (days)	Mean 37 Median 31 Range [22-65]	
	Delay between 2 sessions (days)	Mean 54.6 Median 61 Range [30-92]	
Severe retinal complication (retinal detachement or retinal fold)	0		

## Discussion

Out of 19 children with IP, more than one third presented a retinal involvement, including 3 patients with bilateral lesions, diagnosed at a median age of 19 days. All benefitted from laser treatment and no case of retinal detachment, retinal fold or vitreous hemorrhage was observed at 3 years. This study shows that a prompt dermatological diagnosis followed by a strict ophthalmological monitoring and early prophylactic treatment of retinal vasculopathy prevents blinding complications of the disease.

The main goal of the ophthalmologist in IP is to prevent retinal detachment, which has an incidence of close to 20% in published studies and a very poor functional prognosis [3, 7]. Our work suggests that retinal detachment in IP merely results from the early retinal vasculopathy, if left untreated [4]. Laser therapy is known to efficiently prevent complications of retinal ischemia, including in IP retinopathy [4, 5]. In Chen et al. retrospective series on 25 patients with IP (median follow-up duration = 9.3 years; 0.5–22.8), however, 3 of the 4 youngest patients who had received prophylactic laser developed subsequent tractional detachment [7]. Treatment was realized between 1 month to 2 years of age, later than in our series where laser was performed between 22 and 65 days of life. This can be related to the significant difference between the median age at first retinal evaluation: 11 months in Chen’s series, 25 days in the present one. It is likely that, like in retinopathy of prematurity, laser efficiency requires very early intervention, at the stage of isolated retinal ischemia and before any tractional complication occurs.

There is currently no standard recommendation for ophthalmological screening and follow-up in IP. Two distinct algorithms have been proposed in the literature. Holmstrom and Thoren [8] suggested that screening should occur as soon as possible after birth, then monthly until 3–4 months, every 3 months until age 1, twice a year until age 3, then annually throughout childhood, which is close to our algorithm. O’Doherty et al. suggested a less tight agenda in the case of initially normal-appearing retina; assuming that if normal at first examination under anesthesia, the retina would remain so afterwards [9]. In our study, however, in two cases, initial examination in the clinic was normal, while lesions appeared on second examination. It is now acknowledged that peripheral retinal screening in newborns should be performed by a trained examiner, but should not request systematic general anesthesia, which carries specific risks in infants with IP [10].

One or two laser sessions were necessary to control the disease. New lesions could appear in initially healthy areas, which stresses the need for a strict surveillance after initial treatment. The parameters and efficiency of laser treatment in IP are mainly based on the large existing studies and current recommendations on retinopathy of prematurity (ROP) [11]. Intravitreal injections of anti-vascular endothelial growth factor (VEGF) have recently been proposed as an alternative treatment in IP retinopathy, either as an adjunct therapy [12, 13], or as a first line treatment [14]. As infants with IP may present cerebral vascular involvement and strokes –a theoretical contra-indication for anti-VEGF injections–, and as blood-retinal barrier is not considered mature at the age of treatment, such an option should probably be reserved as a second line therapy for severe and atypical cases [15]. As has been the case in the past for ROP, it is likely that current treatment algorithms are more aggressive than needed [6]. Future studies are needed to help better define the indications of laser treatment in IP, based on a better understanding of the pathophysiology of the vascular involvement.

The study has limitations. This is a small retrospective study on a rare disease. The number of patients prevents any definitive conclusion regarding the possibility of atypical evolutions. Because no supplementary exam was added to our usual standard clinical procedures, infraclinical retinal vascular lesions have certainly been overlooked.

## Conclusions

*Incontinentia pigmenti* appears to be an excellent indication for early systematic screening by ophthalmologists, since retinal manifestations are frequent and initially asymptomatic, the risk of severe complications is high, and preventive laser treatment appears to be efficient when performed on time.

## Data Availability

All data generated or analyzed during this study are included in this published article.
